# Understanding the Wide-Reaching Impact of Healthcare Merger and Acquisition Activity

**DOI:** 10.34172/ijhpm.2023.8049

**Published:** 2023-11-26

**Authors:** David B. Burmeister

**Affiliations:** Department of Emergency and Hospital Medicine, Lehigh Valley Health Network, University of South Florida Morsani College of Medicine (USF), Allentown PA, USA

## Introduction

 Over the past decade, US healthcare institutions have increasingly relied on diversification strategies to promote market growth and to improve financial solvency. The most prominent strategic method utilized during that period has been adding scale to organizations, thereby providing them with larger patient population to care for. Many organizations have deployed tactics like expanding their healthcare insurance product, collaborating with commercial insurance companies, and increasing service differentiation by improving customer service, brand identity, and reputation. While each of these maneuvers has benefits, healthcare institutions have primarily relied on merger and acquisition (M&A) activity, which has become increasingly prevalent across the country. Caring for a larger patient population allows organizations to absorb a larger number of revenue “hits,” expand opportunity for cost efficiency, improve service differentiation, allow for greater control of social determinants of health, and offer increased leverage against government or commercial payers. Healthcare M&A is vital for consistent care and financial viability. Consequently, there are important questions to contemplate:

1. How does this activity affect the marketplace and our society as a whole?
2. How does this activity limit competition and what effect does it have on our regulators?
3. How does it affect our organizational leaders and decisions about how our organizations are structured?
4. How does this activity affect the vital resource of caregivers in our community?
5. What impact does this activity have on the quality of care provided to our patients?

## Marketplace and Society

 The marketplace should rightfully expect healthcare M&A activity to garner value for both the community and the organization. In many cases, the true value for the community is the ability for the local hospital to stay solvent and continue providing care. Unfortunately, this intrinsic value is difficult to identify, as numerous studies have indicated mixed results of M&A activities and their overall value.^1-4^ Some studies have demonstrated less value for the acquired and parent organization, while others have shown improvement in value for only the acquired organization.^4-6^ In many cases, M&A activity can stress the parent organization, often inducing negative effects from both the immediate and long-term perspective.^6-10^

 M&A activity exerts influence on the marketplace and is a vital activity for healthcare to thrive in the future and become more efficient, more patient centered, and improve outcomes. There are a variety of factors that are important to evaluate prior to initiation. Some studies have identified the activity causes a degradation of quality metrics post-transaction.^5-11^ The activity can adversely affect quality metrics, but it also has the potential to improve the health of the community. There is a trend suggesting a degradation in outcomes related to patient satisfaction, but for other metrics such as readmissions and mortality, there is no consistent positive or negative trend throughout the M&A process.^2,4,5,12^ While operational efficiency may improve, expenses have also been shown to increase following these transactions. On balance, it appears that M&A activity increases the cost to patients and our healthcare system.^6,10,13^ Therefore, there is potential that this activity fuels an environment where we are continuously, and with increasing difficulty, challenged to control costs.

 Further compounding the risks imposed by rampant M&A activity is the fact that over-utilization and pricing may be pushing us past a point of no return. It has been noted that medical pricing increases between 20%-45% following M&A activity.^6^ Economists consistently agree that if we continue with the current pricing trajectory the healthcare industry will be more challenged in the near future.^14^ This raises the question: if aggressive M&A activity continues, how can we combat the increase in healthcare costs? This question becomes more important when we acknowledge that 20%-30% of healthcare organizations are actively seeking M&A activities. Health systems have implemented price and quality transparency tools to mitigate negative ramifications.^15^ There are also other important quality outcomes that can be benchmarked to like sized organizations to ensure a high standard of care.

 One of the most important objectives of M&A activity is to improve productive efficiency, thus decreasing costs. At the same time, it is important to address allocative efficiency so that we are maximizing healthcare access for that specific patient population. More aggressive clinical integration early on is the answer and analyzing efficiencies in advance can create a roadmap for greater success providing more value to the organization and to the community.^16^ Shifting hospital-based services to practice-based services, alignment of service lines, consolidating services, sharing infrastructure, and utilizing a similar administrative structure are key considerations. There is significant hospital and service heterogeneity within our healthcare system that has caused disparities in healthcare access. M&A activity allows us to decrease the hospital heterogeneity amongst regions to provide a higher standard of care across a population. Focusing on clinical integration, it gives organizations a better chance of providing more service differentiation and offering more specialized and comprehensive care to patient populations that would not have otherwise had the chance to experience these benefits.

 In rural areas, there is evidence of major consolidation of resources upon acquisition.^4^ This can cause effects on specific specialties within a hospital merger. Over the past years there has been more focus for entities to acquire highly profitable specialties.^17^ Examples of this would be the targeting of ambulatory surgical centers, orthopedic specialty hospitals, cancer resources, and heart and vascular assets. M&A activity may not result in the expansion of services. Many times, we observe consolidation of services with resultant closures or repurposing of entire hospitals, buildings, and practices, which from a management and cost containment perspective has strong support that it would be advantageous to the organization.^4^ Since 2010, there have been hundreds of rural hospital closures. When these rural hospitals are acquired, there are often significant reductions in services for on-site diagnostic imaging, availability of obstetrics and primary care services, and other outpatient non-emergency services.^2,4^ In many cases emergency services are also consolidated or closed. While numerous services are lost in many of these transactions, there is evidence of significant improvement of operating margins for these organizations.^2^

 Health system expansion though M&A activity may also carry clinical risks that impact patient safety. Therefore, extra care must be taken when evaluating the feasibility of M&A activity from a clinical perspective. To do so emphasizes the importance of understanding vital baseline quality outcomes. Additionally, organizations must consider changes in patient populations, quality infrastructure, and the quality of clinicians that are present to better predict what challenges will be faced after the acquisition takes place. When these concerns are evaluated comprehensively and the correct countermeasures are put into place, acquired hospitals may see a reduction in mortality and improvement in quality of care.^2,12^ However, this reality is frequently not observed, as clinical integration is often only considered in the later periods of M&A activity.^16^

## Regulators and Governmental Control

 Regulation of M&A activity by federal and state governments is challenging for those involved, but independent evaluation is necessary. The US Department of Justice (DOJ) and Federal Trade Commission (FTC) had previously developed Integration Guidelines, but those guidelines were recently retired while they redesign the evaluation process. The decision to rescind these guidelines was largely due to the belief that they were too lenient and did not consider impacts on the labor market.^18^ The DOJ and the FTC function on the premise that competition lowers costs for our patients and helps improve innovation, quality of care, and patient experience.^18,19^ The doubling of merger activity from 2020 to 2021 has placed both the DOJ and FTC on “high alert” and has made it difficult to enforce these principles.^20^ The healthcare industry and others are perceived as becoming more concentrated and less competitive; thus, the new measures are meant to strengthen enforcement against limiting competition. This is primarily driven by the potential hazard to quality of care and the DOJ’s belief that they must protect consumers.^21^ Due to the increased activity, revised integration guidelines, and a goal of providing new direction to healthcare entities, there are multiple “checkpoints” that have been instituted along the M&A path:

- Early terminations, will no longer be granted by the FTC.^22^
- Approval will be determined by the value of the transaction plus the amount of certain organizational debts.^23^
- The FTC will expand “Second Request” requirements, forcing companies to include information related to the transactions’ labor market impact and the involvement of investment firms.^24^
- The FTC will increase their use of “Warning Letters,” indicating that mergers may continue “at their own risk” if a review of the transaction has not been completed.^25^
- The FTC will now require parties that recently settled merger investigations to notify the FTC prior to future acquisitions. This was recently seen in a case involving DaVita, where they must seek FTC approval prior to acquisition of any dialysis centers within Utah for the next 10 years.^26^

## Organizational Impact

 The challenge that the administrative hierarchy has with M&A activity revolves around evolving their leadership structure to ensure the entities are managed appropriately. In many cases the existing local management structure could remain if their vision and culture were aligned. Balancing current business due diligence can be challenging, and a lack of focus can impact both current and future operations of the parent organization. The parent organization should define the leadership infrastructure needed to facilitate the acquisition while being careful not to usurp resources that are vital to the success of the current operation.

 The organization needs strict focus on structure when discussions of these transactions begin. A “hands off” management approach to clinical integration is not prudent in any scenario and will negatively affect long-term outcomes and profit margins.^16^ At the same time, how we communicate M&A activity both within and outside the organization should be coordinated, consistent, and timely. The greatest resource any entity has is their people, therefore the execution of the communication cascade needs to be strategically determined. There needs to be accountability of the parent organization to take ownership of the acquired operational and clinical entities. For successful integration, the existing operational and clinical leadership should have completed the planning phase and be prepared to assume immediate ownership of the new entity once the transaction has been finalized.

## Avoiding Past Mistakes

 The American healthcare system is challenged to strike a balance between either a business or service ethic. Over the past 25 years, many have realized how our economic mindset and corporate competencies need to be sharpened for us to fulfill our healthcare mission. The healthcare system has continued to shift from a not-for-profit to a for-profit mindset and has become a multi-trillion-dollar business with opportunity for investors to increase profits and efficiency. This typically low margin business model continues to be challenged with new and unforeseeable clinical scenarios, like the COVID-19 pandemic. An optimized service component becomes increasingly difficult while we try to pull as many dollars as possible into or out of the system. However, regardless of a not-for-profit or for-profit mindset, the fundamental concept of no margin - no mission still applies.

 The dilemma between a business and service ethic should not be considered adversarial. It is the reality of the future. As M&A activity continues to increase, it will be vital for healthcare systems to become more clinically integrated to increase both throughput and efficiency. Without these streamlined workflows, revenue losses will outpace any potential financial gains, thus threatening the financial stability of the acquiring organization. Ultimately, this could lead to a contraction of available healthcare services and impact important quality metrics such as readmissions, inpatient length of stay, mortality, and hospital acquired infections. As value-based care becomes increasingly important to the healthcare revenue stream, the discussion and evaluation of clinical integration needs to start on the day negotiations begin.

## Acknowledgements

 The author would like to thank Peter Rising, and Drs. Kevin Weaver and Bryan Kane for reviewing the article and providing helpful feedback. The author would also like to thank Nicholas S. Imperato, BS, and Katelyn McLain, BS, for editing, formatting, and submission assistance.

## Ethical issues

 Not applicable.

## Competing interests

 Author declares that he has no competing interests.
